# Connecting Home-Based Self-Monitoring of Blood Pressure Data Into Electronic Health Records for Hypertension Care: A Qualitative Inquiry With Primary Care Providers

**DOI:** 10.2196/10388

**Published:** 2019-05-23

**Authors:** Sarah Rodriguez, Kevin Hwang, Jing Wang

**Affiliations:** 1 Cizik School of Nursing The University of Texas Health Science Center at Houston Houston, TX United States; 2 McGovern Medical School The University of Texas Health Science Center at Houston Houston, TX United States; 3 School of Nursing The University of Texas Health Science Center at San Antonio San Antonio, TX United States

**Keywords:** patient-generated health data, connected health, remote monitoring, electronic health record, hypertension, patient reported outcome, self-measured blood pressure, self-monitoring of blood pressure

## Abstract

**Background:**

There is a lack of research on how to best incorporate home-based self-measured blood pressure (SMBP) measurements, combined with other patient-generated health data (PGHD), into electronic health record (EHR) systems in a way that promotes primary care workflow without burdening the primary care team with irrelevant or superfluous data.

**Objective:**

The purpose of this study was to explore the perspectives of primary care providers in utilizing SMBP measurements and integrating SMBP data into the clinical workflow for the management of hypertension in the primary care setting.

**Methods:**

A total of 13 primary care physicians were interviewed in total; 5 in individual interviews and 8 in a focus group. The interview questions were centered on (1) the value of SMBP in hypertension care, (2) needs of viewing SMBP and desired visual display, (3) desired alert algorithm and critical values, (4) needs for other PGHD, and (5) workflow of primary care team in utilizing SMBP. The interviews were audiotaped and transcribed verbatim, and a thematic analysis was performed to extract overarching themes.

**Results:**

The primary care experience of the 13 providers ranged from 5 to 35 years. The following themes emerged from the individual and focus group interviews: (1) ways to utilize SMBP measurements in primary care, (2) preferred visual display of SMBP, (3) patient condition determines preferred scheduling of patient SMBP measurements and provider’s preferred frequency of viewing SMBP data, (4) effect of patient condition on alert parameters, (5) location to receive critical value alerts, (6) primary recipient of critical value alerts, and (7) the need of additional PGHD (eg, emotional stressors, food diary, and medication adherence) to provide context of SMBP values.

**Conclusions:**

The perspectives of primary care providers need to be incorporated into the design of a built-in interface in the EHR to incorporate SMBP and other PGHD. Future usability evaluation should be conducted with mock-up interfaces to solicit opinions on the optimal alert frequency and mechanism to best fit the workflow in the primary care setting. Future studies should examine how the utilization of a built-in interface that fully integrates SMBP measurements and PGHD into EHR systems can support patient self-management and thus, improve patient outcomes.

## Introduction

Poorly controlled hypertension has been shown to increase the risk for cardiovascular diseases and other related deaths. In the United States, approximately 1 in 3 adults and 2 in 3 older adults have hypertension, but only half of these patients have their blood pressure (BP) under control [1]. This presents a significant public health challenge, particularly in the primary health care setting. For this reason, effective intervention strategies that can help patients achieve optimal BP control have become a priority in health care [1]. Appropriately collecting and acting on patient-generated health data (PGHD), such as self-measured blood pressure (SMBP) data, has the potential to better engage patients in self-care, improve patient outcomes, and reduce health care costs related to readmission and emergency room visits. A recent study, implementing a 30-day program that utilized remote monitoring of SMBP measurements, showed significant reductions in the BP values of hypertensive patients [2]. A 2013 systematic review and meta-analysis previously demonstrated the use of SMBP was found to be effective with or without any additional support [3]. However, a more recent systematic review and meta-analysis shows strong evidence that the intensity of additional support combined with the self-monitoring drastically improved the effectiveness of SMBP on lowering BP in hypertensive patients when compared with self-monitoring alone [4]. Additional studies have explored patient and provider perspectives on using SMBP to identify best practices for using SMBP in a clinical practice. Although these studies explored the use of SMBP in clinical practice, no efforts have been made to integrate SMBP directly into the electronic health record (EHR) to fit workflow in the clinical setting. One study examined wirelessly transmitting SMBP measurements to an interactive Web-based system, which is then linked to clinicians’ EHR system [5]. This 6-month pre- and postevaluation study showed promising results in patient BP control. Measuring SMBP with standardized machines that are wirelessly connected to EHR systems yields more objective data compared with the values reported by patients. Combined with other PGHD, including lifestyle behaviors and medication adherence, SMBP measurements can facilitate better behavior changes in patients by providing them with a clear picture of how medication and lifestyle play a role in BP control. The combination of PGHD and SMBP measurements also allows providers to see if the prescribed treatment regimen is having the intended effect. However, 1 challenge that remained from this study was how to further integrate SMBP data into EHR systems and standard clinical workflow. Our study is positioned to address this challenge. The purpose of our study was to explore how primary care providers currently utilize SMBP measurements in their practice and their preferences for a built-in system that integrates patient SMBP data and additional PGHD into the EHR to better facilitate workflow in the primary care setting.

## Methods

### Study Design

A qualitative descriptive study was conducted to explore the insights of primary care providers on the utilization of SMBP in the management of hypertensive patients in the primary care setting. A total of 5 individual interviews were conducted to obtain candid responses from the primary care providers without peer influence. One focus group, with 8 different primary care providers, was then conducted to seek insights from the providers and observe group interaction dynamic on their responses on this topic [6]. The same open-ended questions were utilized in the individual interviews and the focus group interview.

### Participant Recruitment

The study participants were recruited from a local academic health center with multiple primary care sites. An invitation containing only the study purpose was sent by the senior author (JW) via email to solicit participant interest from the Departments of Internal Medicine and Family Medicine. Those who expressed interest were approached to schedule a phone call or face-to-face interview. No data were obtained from those who did not respond to the invitation email. Before this study, participants had no relationship with the senior author.

### Provider Selection

A total of 13 primary care providers participated in the study. All providers specialized in internal medicine, and the primary care experience of the providers ranged from 5 to 35 years. The number of patients the providers saw each month ranged from 49 to 256 patients, with the average being 118 patients. One additional provider had expressed interest in the study but was unable to participate because of loss of follow-up.

### Interview Protocol

Interview questions (Textbox 1) were open-ended and designed to illicit detailed information on how providers currently use SMBP measurements in the primary care setting and their preferences for how they might like to see SMBP data and PGHD integrated into the EHR to facilitate workflow in the primary care setting. The questions were centered on (1) the value of SMBP in hypertension care, (2) needs of viewing SMBP and desired visual display, (3) desired alert algorithm and critical values, (4) needs for other PGHD, and (5) workflow of primary care team in utilizing SMBP. Questions from a previous study that developed and tested the connection of mobile and wearable data to an EHR system for diabetes patients were used as a guide when developing our study’s interview protocol [7]. When necessary, probing questions were used to encourage the participants to elaborate on their responses.

**Interview questions.**

1. What is the value of viewing patients’ home-based monitoring of blood pressure data?
2. How does it influence the care you provide?
3. What blood pressure monitor used—type of monitor, frequency, and time points used?
4. Would you prefer an alert? How should the alert be presented?
5. What do you think the algorithm of the alert should be? What are the parameters to be considered critical?
6. How would you prefer data to be displayed in the electronic health record (EHR)—graphs, charts, or tables?
7. What other patient-generated health data would you like to know?
8. How would you like it to work with the primary care team? Should the information to go to the team? How should they act on it?
9. What do you think would be the impact on workflow and care provided?

Interviews were held in person or over the phone to accommodate the providers’ work schedules. All interviews were audiotaped using a digital recorder and held in an office at a university, including those held over the phone. The interviews lasted between 15 to 40 min. To ensure data consistency, all interviews were conducted by the senior author of this study [6]. During the interview process, no field notes were taken, and no other persons were present other than the moderator and the participant (or participants). Dr Wang’s previous involvement in several focus group qualitative studies on patient self-management of diabetes and obesity using mobile and connected health technologies made her the most qualified of the researchers to conduct the interviews. She had training on focus group methodology. At the time of this study, she was an associate professor at University of Texas Health Science Center—Cizik School of Nursing.

### Data Analysis

Field notes were made after each interview. The audio recordings of the individual interviews and focus group were then transcribed verbatim by a professional transcription service company. The transcripts were not returned to the participants for comment or correction, and no repeat interviews were conducted. A total of 2 members of the research team (SR and JW) utilized conventional content analysis to independently code and derive themes from the transcribed interviews [8,9]. Microsoft Excel was used to code provider responses based on the derived themes that were identified from analyzing the interview transcripts. Discrepancies in theme analysis or coding were resolved via discussion between the 2 members of the research team and verified by repeated cross-checking within and across all transcripts. Data saturation was reached, and the overarching themes that emerged from the individual interviews and focus group, along with supportive quotations, are presented in the Results section.

## Results

### Sample Characteristics

The participants were 13 primary care providers whose primary care experience ranged from 5 to 35 years; all providers specialized in internal medicine. The providers have currently been utilizing SMBP in the management and diagnosis of hypertensive patients and suspected hypertensive patients, respectively. A total of 7 themes emerged from the individual interviews and focus group. Each is discussed below and substantiated by quotes from the transcribed interviews.

### Ways to Utilize Self-Measured Blood Pressure Measurements in Primary Care

The majority of the primary care providers consider home SMBP an important tool in differentiating between patients who suffer from sustained hypertension and those who have white coat hypertension. Providers also mentioned they use SMBP to initiate or titrate medication and monitoring patient condition. Though a few providers raised the issue of reliability or accuracy of SMBP data because of BP cuffs not being calibrated correctly, 2 providers stated this issue can be easily fixed by asking the patient to bring their home BP machine into the office for examination:

[SMBP] helps me to differentiate between an elevated blood pressure due to stress or

white coat hypertension versus consistently elevated blood pressure so I get a

better idea of whether my treatment is working.

Provider 2

[SMBP] influences it a lot because a lot of my patients have white coat hypertension, so they have very high blood pressures...But if I put them on medicines then they end up being hypotensive at home and getting sick.

Provider 5

To make sure we’re on the same playing field, I do have my patients bring in their home blood pressure device and...compare it to what we may get here in the office.

Provider 3

### Preferred Visual Display of Self-Measured Blood Pressure Data in Electronic Health Record

The majority of primary care providers preferred all SMBP measurements taken by the patient be displayed in the EHR as a table. However, they each wished to extrapolate different information from the table. One provider did not have a strong preference for how the data were presented in the EHR, whereas another provider wished to view the data as a graph. Other suggestions for the visual display included color-coding patients’ SMBP measurements based on the Eighth Joint National Committee classes or by default parameters set by the provider:

Yeah, the date, the time for this, if they are checking it multiple times of the day I can correlate that. Then I'd probably want the numbers and then the weekly averages.

Provider 2

Something like a run chart...where you have...the date and time and the blood pressure and it would show like a distribution and you would be able to see from the mean and the standard deviation...I think that would be the most useful to me.

Provider 5

I don’t have any strong preference [as long as the information is there]...and understandable.

Provider 4

If we have the numbers, we can make a graph out of it. If they’re entered as discrete data points.

Provider 8

I was just going with the [Joint National Committee] eight classes and ranges and designating a color for that is a good idea.

Provider 3

### Patient Condition Determines Preferred Scheduling of Patient Self-Measured Blood Pressure Measurements and Provider’s Preferred Frequency of Viewing Self-Measured Blood Pressure Data

The majority of primary care providers mentioned their preferences on how frequently and when patients should take their SMBP measurements. However, it was evident from their responses that the patient’s condition significantly impacted the time of day the SMBP measurements should be taken, the number of SMBP measurements they wanted per week, and how often they wished to view the results of the patient’s SMBP measurements. The preferred frequency of notifications for newly imported SMBP data into the EHR varied across providers and were dependent on the severity of the patient’s SMBP measurements. Provider preferences for viewing SMBP data for well-controlled patients ranged from once every 2 to 3 weeks, once every 3 months, to once every 6 months. Opinions for monitoring uncontrolled or severe patients also varied. Provider views ranged from every couple of days, every week, to every 2 weeks:

Once a day is fine just as long as it's consistent. It may be even variant amongst times throughout the day...Most patients take their anti-hypertensives in the morning so maybe an hour after taking a blood pressure medicine one day and then the next day, maybe an afternoon or the following day, the evening, so we can get what the blood pressure is like across the day and try to get a better assessment that way.

Provider 3

I would probably say like 3 times a week...at different times, like maybe twice, once in the morning and once in the evening, and once after a stressful day or something that they know was a stress trigger.

Provider 1

Poor control, I said three times a week. If it’s more controlled, I might want it twice a day for ten days, but then less after that.

Provider 4

If it’s not...out of range, I would probably say once in two to three weeks.

Provider 1

If it’s severe range and we’re still working to get the blood pressure down, I actually wouldn’t mind being tasked more frequently, like every two to three days.

Provider 3

It depends on how well they’re controlled...If it’s severe range, asymptomatic blood pressure, I am seeing those patients every two weeks. If there’s someone that is just a few points above...Maybe every visit when I am seeing them every 3 months or so.

Provider 3

### Effect of Patient Condition on Alert Parameters

The majority of providers had a set of general parameters for SMBP measurements that would trigger an alert in the EHR. However, the type of parameters and frequency of alerts varied from provider to provider. The suggested values for the alerts for hypertensive readings ranged from 160 to 180 for systolic BP and 100 to 110 for diastolic BP, whereas suggestions for hypotensive alert values ranged from 80 to 100 for systolic BP and 55 to 60 for diastolic BP. One provider did not set a critically high value, but rather, wished to receive a weekly alert if their patient’s average BP readings were out of range of their individually set goal. During the focus group discussion, providers hesitated to provide any parameters for a critical value alert for fear of the possible legal implications they may face if they did not act on a critical alert and a patient were to suffer from an emergency condition such as a stroke. However, a recurrent theme among the focus group participants was how individual patient conditions impacted critical alert parameters:

If your blood pressure is below 100/60 on a consistent basis or above 170/100 please fax or send the information on your blood pressure details immediately. If they're between these parameters send them every week. But if they're less than these then once in two to three weeks, whatever we decide.

Provider 1

If I’ve given them a blood pressure goal of less than 140/90 and their average is above that, then that information is tasked to me...A weekly alert if the average is out of range.

Provider 2

It’s hard to say what a critical value is. There's standards to say what maybe the ideal goal should be, and we can look at national guidelines and say, “This is the goal.” But in terms of what's a critical value, it's really hard to say, it depends so much on the individual patient.

Provider 6

Itdepends on how we fit in the consent form to the patients, about what's their expectations about how physicians or nurses will act on those data. I think as of right now we are trying to limit the liability of [the physicians].

Provider 11

[The button] is grey if it’s someone that’s not using it,...green if their averages are all within the parameter, [and] yellow or red if they’ve got values outside [the parameter].

Provider 2

### Location to Receive Critical Value Alerts

The majority of providers preferred the alert for critical SMBP values appear in the task list of Allscripts, which is the EHR system for the study recruitment site. However, 1 provider requested the alert be sent to their pager instead of the task list because of alarm fatigue and concern about being able to identify, and thus, act upon the critical SMBP value alert in their already inundated task list:

I think the task is fine. I'm looking at that usually just a couple of times a day.

Provider 4

Task is the best way for me.

Provider 3

I would want a page though, not an Allscripts task...It’s alarm fatigue. I have like 50 something messages in Tasks right now and most of them aren’t urgent...

Provider 5

### Team Access to Self-Measured Blood Pressure Data and Primary Recipient of Critical Value Alerts

All providers believed access to SMBP data in the EHR should be available to all members of the primary care team such as nurses, case managers, medical assistants, and other providers in the office. However, opinions differed regarding who should be the primary receiver of critical value alerts. Some providers preferred the alert be sent directly to them first, then they could decide if immediate action is required or if the nurse contact the patient. Others suggested the alerts should be routed to other personnel first, such a nurse, who would then evaluate the data and determine if the provider needs to be notified of the patient’s condition:

I think our medical assistants would be very useful in pulling up the record in, you know, accessing it if it's in some different system and pulling it up in the patient's room so that it's available when we walk in.

Provider 5

I think it should be a part of the medical chart [and] everyone...that is treating the patient [has access to it].

Provider 3

Unless someone is covering for myself, I wouldn’t expect anyone [to act on it]...If you’re tasking me directly with highs...or [SMBP data] out of range...then I wouldn’t expect anyone else to act on it.

Provider 3

Most likely what I would do...if get I these tasks...I would...forward [it] to the case manager and ask for their help.

Provider 2

I’m thinking that it should come to a central place and then the nurses look at it...before they route it to the appropriate provider.

Provider 9

Maybe it should go to a nurse who can call the patient. The nurse could easily triage the patient [to] see if they’re symptomatic, [and] if they are, send them to the ER. If they’re not symptomatic, get them to the clinic or call me.

Provider 5

### Additional Requested Patient-Generated Health Data to Incorporate Into Electronic Health Record to Provide Context of Self-Monitored Blood Pressure Measurements

In addition to the BP measurements, the majority of providers wished to include additional PGHD to better understand SMBP measurements. They expressed how difficult it can be to evaluate SMBP data without context and how additional PGHD can provide a better picture of a patient’s situation at the time a particular SMBP measurement was taken. Most providers wanted to include medication adherence with the SMBP measurements. Some providers also wished to include food diaries, weight, monitoring stressors such as any surgery or life events, emotional states of the patients, alcohol consumption if patients are drinking, heart rate, or the option for patients to include any symptoms they may be feeling if an SMBP measurement were above or below the preferred desired BP range. One provider stated he would like to incorporate additional PGHD but, because of concerns of information overload, he only wanted patient symptoms and BP measurements to be recorded:

I would definitely want to know whether they took their medicines.

Provider 5

The time in which they take the medication. Also, depending on certain anti-hypertensives, a heart rate would be good to coincide with the blood pressure. Some medications tend to lower the heart rate so that would be good to know as well.

Provider 3

If they could keep an intake log that includes their salt...and...water intake...what they're eating...and...a little thing to about their emotional state.

Provider 5

If there was any change in their regular pattern. They're going for surgery, or something, which could've impacted [their blood pressure].

Provider 1

I would also want, if possible,...a way for the patient to trigger if they have symptoms. That’s one of the things that’s super useful in an event [to] monitor.

Provider 5

Something else we might benefit [from is]...symptoms suggesting a blood pressure problem.

Provider 4

I guess in a perfect world, yes, [there is other data I would like to know], but...that...may be information overload. I'd rather go with the results.

Provider 4

## Discussion

### Principal Findings

Our study examined how primary care providers would utilize and incorporate SMBP measurements into EHRs to better facilitate workflow in the primary care setting. All the providers interviewed in this study acknowledged the benefits of utilizing SMBP data in the diagnosis, management and treatment, or monitoring of patients with hypertension. Patient condition had a significant effect on providers’ responses in terms of determining both frequency of readings and critical alert parameters, which resulted in various responses from each provider. However, the majority agreed the critical value alert should appear in the form of a task in the EHR, and all personnel caring for the patient should have access to the SMBP data and PGHD in the EHR. Though there was concern from 1 provider about information overload, the other providers stated PGHD, in addition to the SMBP measurements, would be beneficial in understanding the circumstances of an abnormally high or low reading. The majority of the providers also stated they preferred to view patients’ SMBP readings in the form of a table. However, what information was included in the table and what they wished to extrapolate from the data varied among the providers. Additional differences were noted in whether the provider wanted to receive the critical value alert directly or if they thought it should be routed to a nurse first.

### Comparison With Existing Literature

Similar to previous studies conducted on SMBP, the majority of primary care providers in this study typically utilize SMBP to differentiate sustained hypertension from white coat hypertension [10-13]. Additional scenarios for requesting SMBP from a patient, found in this study and in previous ones, included efficacy of antihypertensives or medication adherence and managing or monitoring of controlled hypertensive patients [10-12,14].

In our study, some providers wished to view individual readings, whereas others preferred to view averages. These variable preferences correlate to differences found among UK clinicians, where some clinicians disregarded the first of 3 readings and looked at the other 2 or eyeballed averages [15]. The responses from providers in our study suggest that despite American Heart Association (AHA) recommendations, individual patient condition greatly influences the number of daily and weekly SMBP readings they recommended to their patient. This, again, corresponds to the same UK study where clinicians in both primary and secondary care settings recommended different frequencies and durations of SMBP schedules based on whether the clinicians were trying to diagnosis a patient with hypertension or help manage an existing hypertensive patient [15].

In a previous study, glucose and SMBP data were uploaded to a telemonitoring system outside of the EHR. The nurses would then access the data from the website, transcribe or summarize it, and manually input the information into the EHR. The workflows of the practices in this study were not drastically impacted because they had critical care advanced nurse practitioner managers assigned to collect and evaluate the data. However, the authors of the study postulated practices without these designated nurse managers may find it difficult to implement the use of a telemonitoring system [16].

A recently published systematic review examined 221 studies reported on what factors contributed to the success and/or failure of the implementation of various electronic health tools. Ultimately, the review concluded workflow was the most important factor in determining whether the intervention of an electronic health tool would be successful [17]. Therefore, it is crucial to involve users in the design phase during the development of new electronic health tools such as the one discussed in our study.

Our study aims to address this issue by identifying provider preferences in an effort to design an application that will directly integrate SMBP measurements and PGHD into the EHR which, in turn, could help improve rather than impede workflow in the primary care setting. The participants in our study recognize that the health of a patient is multifaceted and, to provide each patient optimum care, they must consider more than just SMBP measurements. Additional PGHD in the EHR is important for providing context for SMBP data to assist providers in making more informative decisions and taking better actions. However, increasing clinician burden has been associated with the use of EHR [18]. Therefore, when attempting to add more PGHD into the EHR, it is necessary to carefully examine how members of primary care teams plan to share responsibility for responding to this additional data during different phases of patient care. In summary, the challenges in integrating SMBP data into EHR to facilitate hypertension care is 2-fold: (1) Providers must determine what PGHD is most pertinent in the management and treatment of hypertensive patients and (2) Designing a user-friendly patient application that can both obtain PGHD and SMBP measurements and seamlessly integrate that information into the EHR to make clinical workflow more efficient.

### Strengths and Limitations

As more mobile and wearable devices enter the health care market, there is an inherent need to directly integrate PGHD from these devices into EHRs. This is the first study to examine the perspective of primary care providers in the development of a built-in interface that will fully integrate remote monitoring BP data and additional PGHD into EHR systems to fit primary care workflow. There are several limitations to this study. Providers interviewed in this study were primarily physicians; our next step will be to extend interviews to other health care providers or professionals serving hypertensive patients in the primary care setting. We did not collect demographic information of the providers, as we did not believe their demographic characteristics would significantly influence the study findings, but lack of this information may limit the understanding of our study participants to some readers. Furthermore, as mentioned in the Methods section, convenience sampling was utilized to seek participants for this study. Therefore, our sample was not random which, in turn, may limit the representation of primary care physicians in general. Another limitation is that the providers were recruited from 1 academic medical center. The discussion results from the sample regarding BP control values were not compliant with the current practice guidelines on BP measuring schedule, although the providers had a wide range of years in primary care practice (5-35 years).

### Recommendations for Future Research

AHA guideline recommends beginning SMBP measurements 2 weeks after starting or changing a patient’s treatment regimen. The guideline also recommends SMBP measurements be obtained at least 4 times a day; twice in the morning before taking medications and twice in the evening before supper [19]. On the basis of the findings of this study, we summarized a list of recommendations for the development of a built-in interface that fully integrates SMBP data into EHR systems, which include (1) an initial education session to educate the providers about practice guidelines and recommendations regarding BP control; (2) visualization of SMBP in a simple format; (3) general parameters should be set according to AHA or other guidelines. Providers should have the ability to set individualized parameters based on patient condition; (4) a decision support system with a set mechanism to triage the patients based on their SBMP results need to be in place to reduce clinical burden in reviewing raw SMBP data; and (5) all team members should have access to the data. A team approach in integrating SMBP into primary care clinical workflow is favorable.

### Conclusions

In summary, providers valued SMBP measurements in the diagnosis, treatment, and monitoring of patients. They also suggested additional types of PGHD that may provide contextual information of SMBP data which, in turn, would allow them to better manage their hypertensive patients. Provider preferences on SMBP frequency, provider monitoring frequency, and alert mechanisms varied based on patient conditions. Therefore, flexibility in a connected interface to fit the workflow of primary care teams should be explored in future studies. Future usability evaluation should be conducted with mock-up interfaces to solicit opinions on the optimal alert frequency, mechanism, and information flow coordination among primary care team members in proving patient-centered, team-based hypertension care in primary care.

## Abbreviations

AHA: American Heart Association
BP: blood pressure
EHR: electronic health record
PGHD: patient-generated health data
SMBP: self-measured blood pressure
